# Initial symptoms in individuals with Alzheimer’s or mixed Alzheimer’s‐Lewy body dementia are noted at an older age and are more likely to be amnestic in African Americans than Whites

**DOI:** 10.1002/alz.092426

**Published:** 2025-01-03

**Authors:** Quintasha Beamon, Audrey Zhu, James Bena, James B Leverenz, Jagan A. Pillai

**Affiliations:** ^1^ Cleveland Clinic, Cleveland, OH USA; ^2^ Cleveland Clinic Lou Ruvo Center for Brain Health, Cleveland, OH USA

## Abstract

**Background:**

Amnestic initial symptoms are common in Alzheimer’s disease(AD). However initial non‐amnestic (language, executive or visuospatial) symptoms are seen in ∼20% and associated with faster progression. Given racial differences in clinical and biomarker characteristics in AD, we explored if initial cognitive symptoms differed by race of the participants among autopsy confirmed AD, Lewy body pathology (LBP) and mixed pathology (AD‐LBP).

**Methods:**

A retrospective cohort study conducted among 2422 participants with confirmed ADP, LBP, or mixed ADP‐LBP meeting neuropathogy diagnostic criteria in National Alzheimer Coordinating Center (NACC) data from 2005 to 2019. Participants at initial visit had a Clinical Dementia Rating‐Global (CDR‐G) scale ≤1. Due to sparse data in some minorities, analysis was limited to racial groups that had participants with both amnestic and non‐amnestic symptoms. This limited analysis to 93 African American (AA) and 2,299 White participants. Statistical methods including two‐sample t‐tests and Fisher’s Exact tests and Firth’s logistic regression examined the impact of race, age, sex and education on initial symptoms.

**Results:**

Initial cognitive symptoms in AA were noted at an older age compared to Whites (mean[SD] (71[10] vs 69[10] yrs, p = 0.04) and their age at death was older (83[10] vs 81[10] yrs, p = 0.04) across neuropathologies. When the groups were evaluated within neuropathologies, age at onset differences were significant only in mixed ADP‐LBP (71[9]vs 68[10] yrs, p = 0.029) although a similar trend was noted for other neuropathologies. AA participants were less likely to have initial non‐amnestic symptoms than Whites (7.5% vs 23.6% p<0.0001). Similar results were noted when the groups were evaluated within ADP and AD‐LBP(Only 6 AA participants with LBP limited analysis). AA also differed from Whites on initial MMSE (22 vs 24, p = 0.003), CDR‐Sum of boxes (4.5 vs 4, p = 0.047), female% (51.6% vs 39.7%, p = 0.024) and education (14 vs 16yrs, p<0.001), but no difference in APOE ε4 carriers were noted. Despite considering age, sex and education in the regression model, AA had higher odds, 3.06(95%CI 1.51‐7.92) for amnestic initial symptoms compared to Whites.

**Conclusion:**

Initial cognitive symptoms in AA were reported at an older age than Whites in the NACC. Non‐amnestic initial symptoms were less common among AA than Whites.

